# Accelerating the conceptual use of behavioral health research in juvenile court decision-making: study protocol

**DOI:** 10.1186/s43058-021-00112-1

**Published:** 2021-02-05

**Authors:** Sarah Cusworth Walker, Kristin Vick, Noah R. Gubner, Jerald R. Herting, Lawrence A. Palinkas

**Affiliations:** 1 Department of Psychiatry and Behavioral Sciences, University of Washington, Box 356560, 1959 NE Pacific St, Seattle, WA 98195 USA; 2 Department of Sociology, University of Washington, Box 353340, 211 Savery Hall, Seattle, WA 98195 USA; 3 Department of Children, Youth and Families, Suzanne Dworak-Peck School of Social Work, University of Southern California, 669 W. 34th Street, Los Angeles, CA 90089-0411 USA

**Keywords:** Juvenile justice, Criminal-legal, Conceptual research use, Survey methods

## Abstract

**Background:**

The youth criminal-legal system is under heavy political scrutiny with multiple calls for significant transformation. Leaders within the system are faced with rethinking traditional models and are likely to benefit from behavioral health research evidence as they redesign systems. Little is known about how juvenile court systems access and use behavioral health research evidence; further, the field lacks a validated survey measure of behavioral health research use that can be used to evaluate the effectiveness of evidence dissemination interventions for policy and system leaders. Conceptual research use is a particularly salient construct for system reform as it describes the process of shifting awareness and the consideration of new frameworks for action. A tool designed to measure the conceptual use of behavioral health research would advance the field’s ability to develop effective models of research evidence dissemination, including collaborative planning models to support the use of behavioral health research in reforms of the criminal-legal system.

**Methods:**

The ARC Study is a longitudinal, cohort and measurement validation study. It will proceed in two phases. The first phase will focus on measure development using established methods of construct validity (theoretical review, Delphi methods for expert review, cognitive interviewing). The second phase will involve gathering responses from the developed survey to examine scale psychometrics using Rasch analyses, change sensitivity analyses, and associations between research use exposure and conceptual research use among juvenile court leaders. We will recruit juvenile court leaders (judges, administrators, managers, supervisors) from 80 juvenile court jurisdictions with an anticipated sample size of *n* = 520 respondents.

**Discussion:**

The study will introduce a new measurement tool for the field that will advance implementation science methods for the study of behavioral health research evidence use in complex policy and decision-making interventions. To date, there are few validated survey measures of conceptual research use and no measures that are validated for measuring change in conceptual frameworks over time among agency leaders. While the study is most directly related to leaders in the youth criminal-legal system, the findings are expected to be informative for research focused on leadership and decision-making in diverse fields.

Contributions to the literature

- The study fits within the implementation science literature on behavioral health research use and advances the measurement of conceptual research use among organizational leaders.
- The study advances research methods for studying the impact of behavioral health research dissemination methods and interventions in policy and system decision-making.
- The study focuses on an understudied health service sector in implementation science, the youth criminal-legal system.

## Background

The US youth criminal-legal system is under heavy scrutiny for the role it plays in exacerbating the health and economic disparities of communities of color, particularly among Black citizens. In 2018, nearly 750,000 youth were seen by juvenile courts, with 56% identifying as nonwhite [1]. The intersections of race and poverty and the fragmentation of social services at the community level currently make the justice system the de facto service provider for the most health-vulnerable population of youth in our society [2–4]. Increased recognition of this issue is driving calls for system reform to shrink the criminal-legal response [5, 6] and integrate more behavioral health-focused reforms as alternatives to current processes [7–9]. A majority of youth referred to courts will receive orders to participate in social services through diversion or probation orders and may experience brief or long-term incarceration [10]. Juvenile courts retain considerable discretion in when and how to apply these conditions which can have considerable impact on the exposure of youth to legal consequences [11], potential harms from incarceration [12], and access to psychosocial and social determinant-supportive services [13, 14].

Juvenile court leaders are being asked to develop and implement paradigm-shifting reforms in their work. The level of transformation facing this system requires innovation that goes beyond the boundaries of typical justice operations. For example, courts are being asked to consider how research evidence on adolescent development and behavioral health can support redesign of community diversion, legal decision-making, supervision, and placement conditions [7, 9, 15]. If evidence is to contribute to this decision-making, it is likely to be evidence that emerges from diverse scholarly traditions beyond rehabilitative criminology [16]. Effective methods of accessing and applying evidence along with participatory and community-informed strategies will be critical as the criminal-legal system responds to the current reform impetus. This longitudinal cohort study is focused on understanding the current context of evidence use in the youth criminal-legal system, the relationship between research exposure and subsequent changes in decision-maker thinking (conceptual research use, CRU), and the validation of a conceptual research use survey to support implementation of routine use of research evidence and future study of evidence use interventions.

### Measuring research use

Measuring evidence-informed decision making in public policy and systems is a rapidly growing area of study [17–19]. A prominent theory guiding measurement of evidence use builds from a typology first developed by Carol Weiss [20], and expanded upon by later scholars, notably Pelz [21]. The three most commonly cited and studied types of research in this typology include instrumental use, conceptual use, and symbolic/political use. As described by Beyer [22], instrumental use involves applying research in a *specific and direct way*. This may include deciding to adopt and implement a new practice or program, de-implementing an existing practice or program, or changing a policy. Conceptual use involves changing one’s way of looking at an issue, also termed *enlightenment*. Symbolic use is the use of research to legitimize a previously held position and may be most commonly used to justify *political positions*. Despite widespread acceptance of these constructs as useful delimiters of how research is used in policy and decision-making [23], the measurement of these constructs is often inconsistent depending on the study focus.

Use of research measurement has a long tradition within Implementation Science, with conceptual frameworks and measures developed for policy formation [24, 25], public health decision-making [26–28], and direct service implementation of best practice guides and protocols [29]. Use of research measurement derives from conceptual frameworks that are interested in how research evidence is blended with other types of knowledge and information to guide decision-making. For example, in Armstrong et al.’s [26] study of research evidence use among public health decision-makers, the authors found that a mixture of local demographic data and external research evidence was influential in decision-making, with local data predominating in perceived value. The measurement of conceptual research use, specifically, is a key construct in these frameworks. Within implementation science, a survey measure of CRU will advance the field’s ability to measure the impact of research use interventions (e.g., trainings, communication strategies, policy formulation frameworks) on the degree to which research evidence influences real world decision-making processes.

**Fig. 1:**
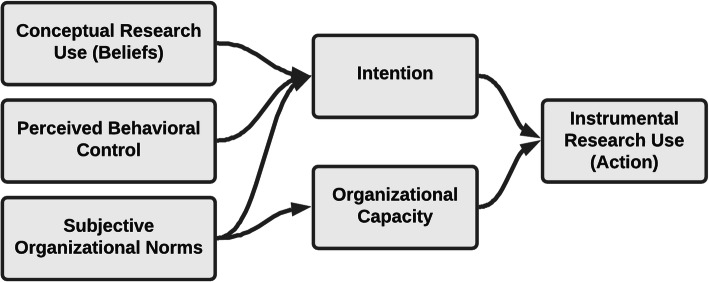
Conceptual model for the study

Conceptual research use is intended to describe transformative changes in thinking (Fig. 1). Unfortunately, of the three uses of research evidence, conceptual research use may be the most difficult to clearly operationalize. The first formulation of conceptual use was Carol Weiss’ [20] description. In this model of use, science findings percolate through various channels to “provide decision makers with ways of making sense out of the complex world.” Policymakers may only rarely be able to cite findings of a specific study but they “have a sense that social science research has given them a backdrop of ideas and orientations that has had important consequences.” Perhaps most importantly, conceptual research use does not assume that “research results must be compatible with decision makers’ values and goals.” Rather, the enlightenment model or CRU, by definition, serves to change or challenge existing frameworks and beliefs. Pelz [21] was first to coin the term conceptual use (while also designating instrumental and symbolic uses) and it has been subsequently understood to represent using research for “general enlightenment” (e.g., Beyer [22]). This has left a fair amount of discretion in how to operationalize the CRU construct in measurement, including simple single-item [30] or multi-item scales [31], qualitative coding of brief or in-depth interviews (e.g., Caplan et al. [32]; see Amara et al. [23] for a review), and content coding from documents [17].

Differences in the measurement of CRU are driven by the diverse interests motivating research use studies in the broader field of knowledge translation and implementation science. A majority of studies that include research use as an outcome are not explicit about the conceptual framework that underlies the researchers’ interest in CRU as a correlate or outcome of other study characteristics [33]. This is critical as the contextual factors surrounding CRU in different systems and different levels of decision making are key drivers of converting CRU to whatever action ends up being most feasible and appropriate. Studies of research use can be grouped at two levels: policy and organizational decision making (collective) and individual [34]. Contandriopoulos provides a theoretical model for collective level research use that highlights the complexity and multiply determined influences on action at this level. They conclude that conceptual use is too difficult to disentangle from symbolic use without direct measurement of cognitive processes.

Contandriopoulos and others have pointed out that the policy and organizational context severely constrain the degrees of freedom available to any individual actor, and recognition of this undergirds most of implementation science [35–37]. A court administrator may be convinced by well-designed research studies, for example, that the use of detention should be limited because of its impact on youth psychosocial well-being, but the administrator’s ability to enact this will depend on a variety of contextual factors outside of her direct control (e.g., political will, prosecutor and judicial preferences, law enforcement practices). Studying instrumental research use alone, such as the adoption of evidence-based practice at a policy or organizational level, does not provide essential information about whether such a decision to adopt was guided by an understanding of its implications. There could be a number of other reasons why evidence gets used in a system that bypasses the preferences of local decision-makers (e.g., state or federal law). In general, this type of evidence-based policymaking, in which decisions are rolled out without sufficient buy-in and understanding, is understood to be counter-productive [38]. Consequently, research on the effectiveness of system and policy level strategies needs to be able to measure the effectiveness of these strategies on raising awareness, motivation and reasoned action as it relates to evidence use, otherwise encapsulated as conceptual research use.

While approaches to the measurement of CRU are fairly prolific, the measurement science of conceptual research use is thin. Existing measures are not well-suited for measuring change over time and are thus limited as outcome measures for understanding the relationship between research exposure and changes in decision making. A systematic review of self-reported conceptual research use utilization in healthcare [29] found 97 unique studies reporting conceptual research use as an outcome. However, no studies reported on the acceptability of the measures, and reliability was only reported in 33% of the studies. We found a single study of CRU measurement that examined multidimensional psychometrics developed for measuring CRU with direct service providers rather than agency leaders so is likely to need modification to be appropriate for measuring CRU for this level of organizational decision making [39]. Using the *Standards for Educational and Psychological Testing* as a framework, the authors examined the response to a five-item Conceptual Research Use (CRU) Scale within a sample of 707 healthcare aides working in 30 Canadian nursing homes. The study found acceptable reliability (Cronbach’s alpha = 0.89) but identified problems in item understandability and conflicting results for a 5-factor (PCA model) vs. 4-factor (CFA model) tool. The authors indicated the need for further refinement of the wording for better understanding and discrimination and the need to test the tool’s sensitivity to change over time.

Since then, qualitative work by Farrell and Coburn [40] has helped to further delineate the components of conceptual research use for leadership decision making. These include (1) the introduction of new concepts; (2) seeing a problem in a new light; (3) influencing the broadening or narrowing of perceived appropriate actions; and (4) providing a framework to guide action. The Squires scale and Farrell and Coburn’s typologies match up well on the introduction of new knowledge and ideas, and in making sense of one’s actions, but Farrell and Coburn also extend the CRU construct by examining the role CRU plays in providing a framework for action.

### Use of behavioral health research in the criminal-legal system

Use of research studies are generally conducted in systems that are presumed to be legitimate and stable (education, healthcare), where the mission of the system is generally agreed upon, and where the primary goal is improvement in practice [36]. These capacities do not currently apply to the criminal-legal system, which is currently under heavy scrutiny around legitimacy and has a history of conflicting mandates (e.g., rehabilitation, punishment). Within the last few years, the National Research Council [8], the RFK Research Council for Juvenile Justice [41], the National Council of Juvenile and Family Court Judges [7], and the National Academies of Science, Engineering and Medicine [42] have all released reports calling for the integration of adolescent developmental science and behavioral health research in justice policy and practice and either directly or implicitly state that inattention to this science will result in policies that harm youth. Our focus on the current practices of the criminal justice system will advance the science of research use by extending the field of inquiry to a unique sector that may be characterized by a wider range of belief systems about the right course of action when compared to other public systems. Further, the small body of research use literature conducted within the justice system is largely focused on police departments [43–49] and legal professionals [50]. For example, in qualitative interviews with judges and attorneys, Jordan and Murphy [51] found that these professionals experienced moments of revelation as a result of research use. Much less is understood about how court systems access and use research knowledge to inform their actions (e.g., use of detention [52]).

### Study hypotheses

This is a fascinating time in criminal justice reform with powerful interests in philanthropy, policy, and grassroots’ organizations advocating against mass incarceration and calling for the integration of developmental and behavioral science within all aspects of justice process, particularly within the juvenile system. Dialogue, political motivation, and incentives are likely to significantly vary by shared values of communities, leading to wide variations in exposure to and use of research. This not only heightens the likelihood of detecting differences in CRU across sites, but also enhances the importance of the study as an effort to document how behavioral health research is being used during a time of significant system transformation. The present study examines the following hypotheses:

- Hypothesis 1. Recent exposure to behavioral health research evidence will be positively associated with an increase in conceptual research use (changing thoughts about a topic area).
- Hypothesis 2. An individual’s self-ratings of conceptual research use will be positively associated with ratings of their conceptual research use by co-working peers.
- Hypothesis 3. Level of conceptual research use will be positively associated with instrumental and symbolic research use later in time.

## Methods

### Study design

This is a longitudinal, cohort, and measurement validation study. The project will be carried out in two phases. The first phase of CRU item selection and refinement will establish construct validity of a CRU measure designed to be sensitive to change. This will be approached using cognitive interviewing and modified Delphi techniques within an item-response theoretical approach. We aim to develop a scale that is unidimensional (CRU) using polytomous scaling (1–5), and will use Rasch analyses to examine item and scale psychometrics. The second phase will capture the longitudinal information needed from juvenile court leaders to explore the relationship between behavioral health research evidence exposure and conceptual research use. Measure validation will be established using analytic techniques widely used to establish validity and measurement of change in healthcare for subjective outcomes [53].

### Study setting and participants

The study will recruit from 80 juvenile court departments and an estimated 800 mid-level managers (judges, administrators, managers, and supervisors) from three western states. We expect to recruit 70% of the courts into the study, resulting in 56 courts and approximately 520 respondents into the sample.

Juvenile court leaders will include all roles within the youth criminal-legal court system involved with decision-making in the operations of the court. This includes judges, administrators, managers, and supervisors and excludes field probation, attorneys, advocates, and law enforcement. The sample is restricted to juvenile court leaders for two reasons. While many other individuals are involved in criminal-legal decisions for youth, juvenile court leaders are key decision-makers who can facilitate or slow reforms through the management of operations and services. Second, study methods include peer ratings of research use among professionals working in the same courts and participants will need to have routine (primary and proximal) contact with each other in order to complete these ratings.

Judges, often along with juvenile court administrators, oversee all service divisions in a juvenile court. This includes the coordination of all programming an adolescent and their family might experience apart from attorney services. This most often includes probation services for youth who are court ordered to services, diversion services for youth who are ordered to services without a court hearing and may include detention, dependency, and other civil services involving youth (e.g., youth or families petitioning the court for assistance for housing or to manage youth behavior). Judges and administrators play a key role in setting policy and programmatic direction for a court and they are often involved in day to day operations. In very large counties, the court administrator may function in a purely administrative role, and day to day management is delegated to other managers. When this is the case, the managers rather than the administrator will be recruited for the study. Probation supervisors are directly responsible for field probation officers and their performance. When innovations are adopted, supervisors are key players in implementation of new practices and they are often involved in suggesting, developing, and managing new practices in coordination with managers and administrators.

### Procedures

#### Construct validation procedures

A construct valid measurement of conceptual research use needs to be clear in describing the pathway of changed and changing thinking specifically attributable to this type of knowledge exposure. In areas where measurement is just emerging, such as CRU, construct validation involves careful construction of items based on the theoretical literature [54] and iterative feedback from experts and intended respondents [55, 56]. Our team will apply all three methods in developing the scale to be used in the cohort study. We will begin with a theory-informed approach, using Knott and Wildavsky’s [57], early, prescriptive model for research use in organizations, Belkhodja’s [58] Determinants of Knowledge framework, and Coburn and Farrell’s qualitative work [59], along with foundational CRU theory from Weiss [20], Pelz [21], and Beyer [22]. Items will be constructed to reflect the language and framing likely to be understandable to juvenile court leaders. We will then use a Delphi method to obtain expert ratings of these items from five notable conceptual research use scholars, using a ranking scale as recommended by Davis [60, 61]. This will involve sending the expert group the initial items, asking them to rate items on a 7-point scale indicating the item’s consistency with the conceptual research use construct (*how well do the following items reflect conceptual research use*, 0 = not at all, 5 = perfectly), and add comments to explain ratings. The range and mean of ratings per item and narrative feedback will be collected and sent back to the expert group. This will be followed by a group discussion and another round of rating items in group during which ratings will be viewed in real time. Items rating below 4 on the scale in this second round will be reworded in the expert meeting.

We will then conduct cognitive interviewing procedures with these items with juvenile court leaders recruited from one of the states in our sample pool. The research team will coordinate with the state professional organization for juvenile court administrators to coordinate recruitment. A minimum of 10 juvenile court leaders will be recruited to participate in a 30-min phone call during which a facilitator will use cognitive interviewing to ask leaders to “think aloud” as they read through the items [55, 62]. These interviews will be recorded, transcribed, and rapidly coded using content coding [63]. Items that emerge as problematic from coding will be reworded by the research team and brought back to the expert group and interviewed juvenile court leaders for additional discussion, final revision, and consensus. We do not anticipate difficulties reaching consensus, but if they arise, final decisions will be made by the study co-investigators.

#### CRU survey data collection

The research team will partner with intermediaries in each state/region to develop a plan for recruiting juvenile courts into the study. This will include informational activities (e.g., webinars, flyers) with recruitment occurring via email. To participate, a judge or administrator at the court will forward a recruitment email to their court leadership. In order to have a sufficient number of respondents per site for statistical analyses, a minimum of three court leaders must enroll in the study for the site to participate. Staff will indicate interest in participating via an online link in the recruitment email, which will begin the online consent process. Participants will complete a survey protocol that includes the CRU measure once a month for 5 months.

### Measures

#### Conceptual research use (CRU)

CRU will be measured with the items developed through the Delphi and cognitive interviewing processes described above. The development of items will take into consideration the items developed for the Squires [39] study as the most psychometrically well-supported measure available. The Squires CRU scale is five items scaled from *never* to *almost always* (1–5). The survey prompt asks respondents to consider their last typical workday and indicate how frequently best practice knowledge influenced the respondents’ understanding of their job (worded as “how often did best practice knowledge about [work-related tasks] do any of the following”). Items include, for example “Give you new knowledge or information about how to care for residents.” The scale demonstrated good psychometric properties for their sample and purpose (alpha = 0.89), and acceptable item factor loadings for a 4-item measure (0.60–0.70).

#### Instrumental and symbolic research use (IRU, SRU)

Items measuring IRU and SRU will be included in the CRU survey to conduct convergent validity analyses and to conduct preliminary formative analyses on the predictive relationship between CRU on later IRU and SRU. These items will also be developed with expert input and adapted from previous measures [39, 64].

#### Organizational readiness to change

Respondents will be asked to fill out the Context and Change Efficacy subscales of the Workplace Readiness Scale [65] at baseline. These constructs measure whether the work climate is generally open to change (e.g., the senior leaders are willing to try new things), and whether the organization has the capacity to change (e.g., we have the skills and expertise and resources to implement a [wellness] program). Items will be modified to suite the court context. The WRS demonstrated acceptable psychometrics with Cronbach alpha scores of 0.83 and 0.75, respectively.

#### Culture of research use in leaders’ organizations

We will use the four item culture of research use scale developed for a previous study of research among educational leaders [66]. The items were found to be moderately correlated with self-reported conceptual use (*r* = .44).

#### Exposure to research

Exposure to research evidence will be measured by items developed by the study informed by Belkhodja’s Determinants of Knowledge [58] framework to assess previous month discussion of behavioral health research-based information. Respondents will be asked to describe the context and substance of these findings including whether the research contained new information, how they heard about it, whether it was a research-based idea or specific study, the originating source of the research (if known), how relevant the research was, and why (the take home message or big idea).

### Ethical considerations

This study has been reviewed and approved by the University of Washington’s Institutional Review Board (IRB; STUDY00010933).

### Analytic approach

Procedures and analyses for establishing the *cognitive coherence* of items are described above. We will examine the CRU measure as a unidimensional construct using Item Response Theory [67] and Rasch analysis for polytomous items [68]. This will involve examining the item to scale relationship to other items and the measure as a whole. We will use STATA 16 to conduct analysis and examine item performance using item characteristics curves (ICC), category characteristics curves (CCC), and test characteristics curves (TCC). We will also conduct Cronbach alpha tests, and test-retest reliability analyses from Classical Test Theory to facilitate comparisons with previous studies. We expect to observe moderate correlations (> .30) between conceptual research use and attitudes towards research as a test of convergent validity.

We will use hierarchical, regression-based models and well-established methods outlined in Stratford and Riddle [53] for testing the responsivity of change measures in healthcare for subjective outcomes, such as client pain levels, to test our three hypotheses.

#### Hypothesis 1: Recent exposure to research will be positively associated with an increase in conceptual research use

Individual change will be tested by comparing two groups of individuals who vary in their exposure to research during the observational timeframe. This is comparable, for example, to a study of the sensitivity of a pain measure to detect higher levels of change in individuals with acute back pain compared to those with chronic back pain. As those with acute back pain are expected to have greater changes in pain over time, a measure’s success in detecting greater changes in this group is indicative of its sensitivity [69]. Similarly, the proposed study will assess the CRU measure’s ability to detect higher CRU change in those with naturally occurring evidence exposure vs those limited exposure. Those with recent research exposure would be expected to experience short term increases in CRU. Observing the expected changes in CRU as a result of these exposures will also provide a test of the measure’s sensitivity [53]. We will use hierarchical linear regression, nesting within individuals, to examine the relationship between previous month research exposure and concurrent CRU score. To examine the relationship between cumulative exposures, we will examine the correlation between average research evidence exposure over 5 months, mean CRU score over 5 months, and mean CRU change scores over 5 months. Finally, we will examine the time-varying sensitivity of the CRU measure using previous month research exposure as a predictor of CRU change 2 months following pre-exposure in a growth model of CRU over time. We expect natural exposure to be positively associated with CRU levels and higher CRU change over time.

#### Hypothesis 2: Self-ratings of conceptual research use (CRU) will be positively associated with agency peer ratings of conceptual research use

Change sensitivity as measured by peers will be tested by examining the sensitivity of the CRU measure to change using external observer ratings from institutional peers who are also participating in the study. Surveys of research use will ask participants to list other midlevel managers within their organization who have discussed the implications of research findings in formal or informal settings within the last month. Analysis will follow techniques adopted by social network modeling to assign individuals a location score based on the number of times they are mentioned by other peers over the course of the study [70]. The CRU measure’s validity will be assessed by comparing the time-dependent measure of CRU in self-report and corresponding peer rating for the same month. This approach is comparable to comparing a physician rating of health with a patient rating of health and is widely used in testing measure validity. This will involve calculating the independent *t* test and bivariate correlation analyses of the mean differences in the average 5 months CRU rating for each individual and their peer nomination rating (summed mentions within and over 5 months divided by the number of raters in the individual’s organization). For example, an individual who had a mean CRU score over months 1 through 5 of the following (3.5, 4.2, 2.5, 4.2, 3.0) would have a total CRU score of 3.5. If the average of seven organizational peer ratings over the 5 months were (2, 3, 3, 5, 4) the individual’s peer nomination score would be 3.4. Change in CRU would be additionally tested by isolating the individual’s highest 1 month increased CRU change in the time period, in this case months three to four (4.2 − 2.5 = 1.7) and corresponding change in peer nomination in the same months (5 − 3) = 2. We expect peer nomination and CRU change to be positively associated.

Study data will be examined for sufficient range/variation and distributional qualities for best calculating or assessing change within and between groups using descriptive and univariate tests. We will calculate the standardized response mean (SPM) to assess the sensitivity of the CRU outcomes measure to change over time, regardless of condition.

#### Hypothesis 3: Prior conceptual research use of a specific idea tied to a research finding will be associated with later instrumental and symbolic use associated with the same idea

Prior statement of CRU related to a particular research finding will be associated with the presence of a later event using that finding in a symbolic or instrumental manner. We will use simple discrete event history modeling where prior CRU at time *t*-*k* is associated with presence of symbolic/instrumental use at time t accounting for covariates and right censoring.

### Power analysis and sample size

Following the guidelines provided by van Breukelen and Candel [71] and assuming low-moderate intraclass correlation [72], as estimated by the correlation between a leaders’ rating of organizational focus on research and their own CRU (*r* = 0.44), we estimate an intraclass correlation (ICC) of 0.10. Based on this, the study needs a minimum of 40 sites with 13 individuals within each site to detect a moderate effect size (*d* = 0.50) in a two-tailed test, or, 80 sites with 5 individuals per site to detect the same effect. Consequently, our projected 56 sites with 9–10 respondents per site are at an acceptable sample range for adequate power. Lower than projected enrollment 1 year into the study will result in assertive recruitment of other systems to ensure adequate sample size.

## Discussion

Measuring the change in decision makers’ thinking following exposure to behavioral health research evidence is a critical step in modeling a path of reasoned action ending in observable action. This is particularly important in policy and systems characterized by conflicting paradigms in which evidence is highly contested and the right actions are not predetermined. This study will provide information about the conceptual use of behavioral health research among juvenile court leaders that will advance the study of research use in this particular arena.

There are some limitations in the study. First, the sample is limited to juvenile court leaders although transformational changes are likely to require buy in and coordination from multiple professionals who have an interest in criminal-legal proceedings (attorneys, judges, advocates, health systems, families, youth). A comprehensive view of how research is accessed and used in these settings will need to include these other actors and will be a useful focus for additional study. Second, our primary sampling plan will oversample from the Western United States which may limit generalizability. This sampling plan allows us to access our existing collaborations with intermediaries who can facilitate court recruitment and engage a sufficient sample size. Despite these limitations, this study will contribute to the field’s understanding of the valid measurement of behavioral health research evidence use and what types of evidence sources and dissemination strategies are related to increased use.

## Data Availability

Not applicable.

## Abbreviations

ARC: Accelerating Conceptual Research Use in Courts
CRU: Conceptual research use
PCA: Principal component analysis
CFA: Confirmatory factor analysis
IRU: Instrumental research use
SRU: Symbolic research use
WRS: Workplace Readiness Scale
IRB: Institutional Review Board
ICC: Item characteristics curves
CCC: Category characteristics curves
TCC: Test characteristics curves
SPM: Standardized response mean
